# Assessing the costs and returns of on-farm food safety improvements: A survey of Good Agricultural Practices (GAPs) training participants

**DOI:** 10.1371/journal.pone.0235507

**Published:** 2020-07-02

**Authors:** Todd M. Schmit, Gretchen L. Wall, Elizabeth J. Newbold, Elizabeth A. Bihn

**Affiliations:** 1 Applied Economics and Management, Cornell University, Ithaca, New York, United States of America; 2 Produce Safety Alliance, Cornell University, Geneva, NY, United States of America; 3 Cornell Cooperative Extension, Penn Yan, NY, United States of America; Shandong University of Science and Technology, CHINA

## Abstract

Good Agricultural Practices (GAPs) training programs were developed to provide guidance to fruit and vegetable growers on how to reduce food safety risks on the farm. These programs have been enhanced over the years due, in part, to increasing buyer and regulatory requirements. However, the costs of implementing additional food safety practices has been identified as a primary barrier to long-term farm financial feasibility, particularly for smaller scale producers. A survey of past participants in New York State revealed that increasing food safety improvements facilitated by GAPs have not significantly impacted the size of farm operations or the types of crops grown. In terms of farm size, we show that both the financial costs and financial benefits of food safety improvements increase with farm size, but at decreasing rates. In so doing, relatively higher market sales gains per acre by smaller farms from additional food safety investments offset the relatively higher costs to them of their implementation. We also demonstrate that benefits of food safety improvements were significantly higher for farms that had third-party food safety audits and for those that market primarily through wholesale channels. The results should prove welcome by educators as they encourage participation by all scales of producers in GAPs trainings and for growers in understanding that food safety investments can support both reduced microbial risks and sales growth.

## Introduction

Fresh produce has been implicated in a number of foodborne illness outbreaks in recent years, significantly impacting the health of consumers and the economic viability of fruit and vegetable farm operations [1, 2]. Ultimately, the continued occurrence of outbreaks and epidemiological data implicating fresh produce as a major cause of illnesses has caused a shift towards more prevalent food safety requirements and regulations in the past decade [3]. Since microorganisms are extremely difficult to remove once they have attached to produce, proper food safety practices at the farm-level are important [4, 5]. Furthermore, the costs incurred to farm operations because of food safety outbreaks in produce are likely far greater than the costs to them in preventing such incidents from occurring [6].

Good Agricultural Practices (GAPs) were developed to provide basic guidance to growers on how to reduce food safety risks on the farm [7]. GAPs can be traced back to the 1998 United States Food and Drug Administration (FDA) Guide to Minimize Microbial Food Safety Hazards for Fresh Fruits and Vegetables [8]. Though this document outlined voluntary guidance to fruit and vegetable growers, many buyers demanded the implementation of GAPs as a purchase requirement. Because buyers required a way to verify growers were implementing GAPs, third party audits (TPAs) have become a common buyer request. To verify that GAPs are in place through a TPA, growers need to develop a written farm food safety plan and verify implemented practices through recordkeeping. Between 1998 and 2011, buyer requirements and the produce industry were the primary drivers of implementation of GAPs on the farm.

In 2011, the passage of the Food Safety Modernization Act (FSMA) Standards for the Growing, Harvesting, Packing, and Holding of Produce for Human Consumption, commonly referred to as the Produce Safety Rule (PSR), introduced the first ever federal regulation pertaining to the growing, harvesting, packing, and holding of produce for human consumption [3]. One anticipated result of the new FSMA requirements was the continued need for growers to have access to educational opportunities to increase their produce safety knowledge, their ability to assess risks and implement GAPs, and to meet regulatory requirements. Even produce growers who are exempt from the final regulation will need access to this information and training opportunities because it is likely that they will be subject to market-specific food safety requirements that align with the FSMA PSR.

The cost of implementation has been identified as a primary barrier to implementing food safety practices on the farm [9–11]. Costs are complex and include the time to develop farm food safety plans, establish recordkeeping practices, hire additional labor, train workers, and invest in additional inputs, supplies, infrastructure, and equipment. Decision-making is further complicated since growers need to weigh uncertain benefits of changing food safety practices against their significant costs [11]. Such benefits may include maintaining and expanding existing market channel sales, accessing new markets and buyers, and strengthening of their farm brand to prospective buyers due to their food safety improvements.

Distributional implications across farms of different sizes regarding the costs of implementing improved food safety practices have received considerable attention. Studies using grower surveys or case studies have consistently found that expenditures on food safety practices increase with farm size but less than proportionally [9, 12–18]. In other words, smaller farms have higher average costs per acre in food safety investments relative to larger farms. Market equilibrium models incorporating foodborne illness outbreaks that simulate long-term market effects have also demonstrated large growers will benefit more from FSMA relative to small growers [19]. The results of these studies have caused concern regarding the continued financial feasibility of smaller farms given the relatively larger cost burden they face in meeting increasing food safety regulatory requirements. However, potential sales benefits from improving food safety practices on farms have been ignored, as has the ratio of benefits to their costs to which the ultimate question of relative burden should be considered.

The purpose of this paper is to contribute to a more complete understanding of what’s required by producers in making food safety investment decisions. In doing so, we make three important contributions to the scientific literature. First, we examine explicitly the potential benefits along with the costs of food safety investments by farm size. To do so, we expand on the decision model of Adalja & Lichtenberg [18] to assess changes in both expenditures and sales as a direct result of food safety improvements.

Second, we rely on a unique data set from GAPs training participants in New York State (NYS) that enumerates additional food safety investments made by producers as a result of the GAPs training received and the costs and benefits to producers from those investments. In so doing, we are able to explicitly account for changes in food safety costs relative to changes in food safety benefits, as opposed to most of the literature that examine the costs of existing food safety practices already in place [9, 14, 16–18]. GAPs programming evaluations have been conducted in other regions [20, 21], but none formally considered the costs and returns to growers of food safety improvements as a result of the training programs.

Finally, our research will importantly serve as a guide to and encouragement of future food safety training efforts as to its relevance to farms of all sizes and marketing practices. This research will help alleviate the fear for some growers of what food safety improvements might cost and allow them to focus on risk reduction. Notably, as part of our assessment, we evaluated if food safety concerns are causing fruit and vegetable growers to reduce or eliminate production of horticultural crops, and shift production towards other crops such as agronomics which may present lower food safety risks (e.g., corn, soybeans). Maintaining a supply of domestically grown fruits and vegetables is important to consumers that prefer to buy locally grown crops but could also be seen as a national and international priority to make sure consumers have access to safe, fresh produce [22].

We continue with a discussion of the conceptual framework for this research and the materials and methods employed to address our objectives. The empirical results follow, including both descriptive statistics of the costs and returns from our sample of fruit and vegetable producers and regression results that identify changes in costs and benefits of food safety improvements by farm size. We close with conclusions and implications of our results, along with suggested directions for future research.

## Conceptual framework

Expanding on the decision model of Adalja & Lichtenberg [18], we are interested in how the costs and benefits of implementing new food safety practices vary with respect to farm size (acreage). Consider a grower using existing production inputs **X** and food safety practices **Z** to produce outputs **Y** on a farm of fixed acreage A and farm characteristics **D**. The grower maximizes profit:

π=pY−wX−rZ−F (1)

where **p**, **w**, and **r** are vectors of output prices, input prices, and food safety practice costs, respectively, that are assumed constant across growers, and **F** is a vector of fixed costs. The first order profit maximizing conditions (i.e., ∂π/∂Y = ∂π/∂X = ∂π/∂Z = 0), imply optimal use of productive inputs, food safety practices, and output as functions of prices, given existing farm size and farm characteristics; i.e., X^*^(**p**,**w**,**r**;A,**D**), Z^*^(**p**,**w**,**r**;A,**D**), and Y^*^(**p**,**w**,**r**;A,**D**), respectively. Total expenditures on food safety (E) are then E = rZ^*^.

Buyers may require changes in food safety practices (**ΔZ**) for farms, induced by regulatory or other reasons, as part of their contractual relationship. Farms that fail to implement additional practices lose pre-existing farm sales from those buyers, defined as **Y**^**-**^ > 0. However, implementation of new food safety practices may expand sales to new or existing buyers that were previously unattainable given a lack of certain food safety practices, defined as **Y**^**+**^ > 0. Accordingly, the sales volume benefit due to the implementation additional food safety practices is **ΔY** = (**Y**^**-**^ + **Y**^**+**^ > 0). In terms of farm revenue (R), where R = **pY**, the financial benefit of implementing **ΔZ** is **Δ**R = **pΔY**. Assuming that implementation of food safety practices does not affect the optimal use of production inputs **X** at a given level of **Y**, the grower implements new food safety practices when:

pΔY≥rΔZorΔY/ΔZ≥r/p (2)

In addressing how costs of implementing additional food safety practices change with farm size, an expenditure elasticity for food safety improvements (*γ*_*z*_) is derived as:

$$\gamma_{z}=\frac{\partial E}{\partial A}\frac{A}{E}=r\frac{\partial Z}{\partial A}\frac{A}{E}=\frac{\partial Z}{\partial A}\frac{A}{Z}=\frac{\partial Z}{\partial A}/\frac{Z}{A}$$ (3)

where *γ*_*z*_ represents the percentage change in food safety expenditures for a one percent change in acreage. If the marginal change in food safety practices as farm size increases is less than the average per acre use of food safety, then the cost of food safety improvements increases less than proportionally to farm size. In other words, larger farms spend less on food safety improvements per acre than smaller ones. That result is consistent with the literature [9, 12–18] and to which we estimate directly in the regressions that follow.

We are also interested in how the benefits of implementing additional food safety practices change with farm size. Accordingly, using **Δ**Y/**Δ**Z from Eq (2) under equality to approximate ∂Y/∂Z, the revenue elasticity for food safety improvements (*δ*_*z*_) is derived as:

$$\delta_{z}=\frac{\partial R}{\partial A}\frac{A}{R}=p\frac{\partial Y}{\partial A}\frac{A}{R}=p\frac{\partial Y}{\partial Z}\frac{\partial Z}{\partial A}\frac{A}{R}=\frac{\partial Y}{\partial Z}\frac{\partial Z}{\partial A}\frac{A}{Y}=\frac{r}{p}\frac{\partial Z}{\partial A}\frac{A}{Y}=r\frac{\partial Z}{\partial A}/\frac{R}{A}$$ (4)

where *δ*_*z*_ represents the percentage change in food safety benefits for a one percent change in acreage. If the marginal cost of implementing new food safety practices as farm size increases is less than the average revenue per acre, then the benefits of food safety improvements increases less than proportionally to farm size. In other words, larger farms receive less on food safety improvements per acre than smaller ones. That result has not, to date, been empirically tested and to which we estimate directly in the regressions that follow.

As an alternative to considering benefit and cost functions separately, we also consider the ratio of benefits to costs of food safety improvements; i.e., a benefit-cost ratio or BCR = **Δ**R/**Δ**E. Note, a BCR greater than 1 implies the benefits of the food safety improvements are larger than their costs. Here, the BCR elasticity for food safety improvements (*α*_*z*_) is derived from Eqs (3) and (4) and using the quotient rule. Specifically:

$$\alpha_{z}=\frac{\partial BCR}{\partial A}\frac{A}{BCR}=\left(\frac{E\frac{\partial Ry}{\partial A}-R\frac{\partial E}{\partial A}}{E^{2}}\right)\frac{A}{R/E}=\left(\frac{E\delta_{z}\frac{R}{A}-R\gamma_{z}\frac{E}{A}}{E}\right)\frac{A}{R}=\delta_{z}-\gamma_{z}$$ (5)

where *α*_*z*_ represents the percentage change in the BCR for a one percent change in acreage and is expressed simply as the difference of the revenue and expenditure elasticities derived above. It can be computed from estimated values of *δ*_*z*_ and *γ*_*z*_ or estimated empirically on its own as shown below.

## Materials and methods

In the spring of 2014, surveys were administered in person or over the phone to participating growers who had completed the Cornell Cooperative Extension (CCE) and National GAPs Program 2-day GAPs Training and Farm Food Safety Plan Writing Workshop in NYS over the previous five years (S1 File). All of the growers that responded to represented different farms from across NYS. All 350 farms that had participated in the two-day training since 2009 were contacted. Eighty farms fully participated in the survey for a 23% response rate.

The survey was administered to and about farm businesses. Per Cornell University’s Office of Research Integrity and Assurance, the study falls under purposes of organizational effectiveness and, therefore, is not considered human participant research as defined by the Department of Health and Human Services Code of Federal Regulations 45CFR 46. Therefore, the research was not subject to review and oversight by Cornell University’s Human Research Protection Program, and Institutional Review Board approval was not required. Nonetheless, all respondents were 18 years or older and gave oral consent to the survey enumerator before proceeding.

### About the survey

The survey was adapted from a similar survey administered by Colorado State University [17] and included sections about farm characteristics and the costs of implementing various food safety practices. In our survey, a section on the impact on sales as a result of changes in their food safety practices was added. In order to gauge whether or not the operation had changed as a result of implementing GAPs, the survey also asked how many acres were planted in fruit and vegetables, what crops were produced, and if they had conducted a self-audit, both before implementing GAPs (pre-GAPs) and after implementing GAPs (post-GAPs). The survey also asked if there were livestock on the farm, if the farm fields were open to the public, if the farm had completed a TPA, and the farm personnel’s top three reasons for implementing food safety practices.

Costs associated with implementing food safety practices span several dimensions. To address costs associated with staff time, the survey asked for additional expenditures related to training workers on basic hygiene practices, monitoring and inspecting crops for contamination, handling produce, cleaning tools and equipment, recordkeeping, and other tasks related to food safety. With respect to job creation, the survey asked if additional staff had been hired or roles had been expanded to develop or implement GAPs on the farm. To better understand reoccurring costs, the survey asked for the additional annual costs of testing (e.g., irrigation and postharvest water, soil and soil amendments), disposable supplies (e.g., soap, paper towels, gloves, office supplies, traceability supplies, packaging) and any other food safety costs (e.g., new packaging with food safety labels, TPAs, insurance). Finally, the survey asked about one-time and annual investments and improvements made specifically for food safety. This included construction or rental of toilet facilities for workers, creation of food safety policy signage, implementation of wildlife control, changes to water source, changes to raw manure use, storage construction, and harvesting, packing, cooling, and processing equipment purchases.

Beyond costs, growers were asked about the distribution of total farm sales by marketing channel (e.g., direct-to-consumer markets (DTC), distributors, retail groceries, etc.). Finally, the survey asked for the dollar value of sales that would have been lost if they did not implement the additional food safety practices indicated earlier in the survey (**Y**^**-**^, hereafter referred to as maintained sales benefit) and for increased sales to new or existing markets as a result of implementing the additional practices (**Y**^**+**^, hereafter referred to as expanded sales benefit).

### Regression analysis

As a preliminary analysis, average costs, sales benefits, and BCRs were estimated by farm size categories and by whether or not the farms have experience with TPAs. However, to more comprehensively examine changes in costs and benefits, regression analyses were conducted based on the conceptual framework above using SAS Version 9.4 (S1 Code, S1 Data). While our particular focus is on the influence of farm size on food safety costs and benefits, additional control variables were included in the regressions to better identify the independent sources of variation on food safety costs and benefits. Recall from above that X^*^, Z^*^, and Y^*^ are conditional on a given set of farm characteristics **D**. Particular characteristics relevant to food safety improvements pertain to farmer experience with TPAs and their marketing channel choices. Accordingly, we include two indicator variables in the regressions. The first variable, *TPA*, has a value of one if the farm participated in a TPA and zero if it did not. The second variable, *DTC*, has a value of one if the farm primarily markets (i.e., more than 50%) produce through DTC channels and a value of zero if it does not. In addition to identifying more precise measures of elasticities with respect to farm size, accounting for these farm characteristics allows us to identify their impacts on the level of food safety costs and benefits independently.

The natural logarithm (ln) of each dependent variable (*Cost*, *Benefit*, *BCR*) was regressed on the natural logarithm of acreage in fruit and vegetable production. One acre is equal to approximately 0.4 hectares (ha). The resulting form of the regression equations for farm *i* is:

$$\ln\left({Cost}_{i}\right)=\beta_{0}+\beta_{1}\;\ln\left({Acres}_{i}\right)+\beta_{2}{TPA}_{i}+\beta_{3}{DTC}_{i}+\varepsilon_{i}$$ (6A)

$$\ln\left({Benefit}_{i}\right)=\tau_{0}+\tau_{1}\;\ln\left({Acres}_{i}\right)+\tau_{2}{TPA}_{i}+\tau_{3}{DTC}_{i}+\epsilon_{i}$$ (7A)

$$\ln\left({BCR}_{i}\right)=\theta_{0}+\theta_{1}\;\ln\left({Acres}_{i}\right)+\theta_{2}{TPA}_{i}+\theta_{3}{DTC}_{i}+\vartheta_{i}$$ (8A)

where the β’s, τ’s, and θ’s represent coefficients to be estimated, and ε, ϵ, and υ are residual error terms. The estimated coefficients on farm size are directly interpretable as our elasticities of interest; i.e., β_1_ = *γ*_*z*_, τ_1_ = δ_z_, and θ_1_ = α_z_. To assess the independent contributions of *TPA* and *DTC* to the financial impacts of food safety improvements, the equations can be equivalently expressed by taking the exponent of each side:

$${Cost}_{i}=e^{\beta_{0}}{{Acres}_{i}}^{\beta_{1}}\;e^{\beta_{2}{TPA}_{i}}\;e^{\beta_{3}{DTC}_{i}}\;e^{\varepsilon_{i}}$$ (6B)

$${Benefit}_{i}=e^{\tau_{0}}\;{{Acres}_{i}}^{\tau_{1}}\;e^{\tau_{2}{TPA}_{i}}\;e^{\tau_{2}{DTC}_{i}}\;e^{\epsilon_{i}}$$ (7B)

$${BCR}_{i}=e^{\theta_{0}}\;{{Acres}_{i}}^{\theta_{1}}\;e^{\theta_{2}{TPA}_{i}}\;e^{\theta_{2}{DTC}_{i}}\;e^{\vartheta_{i}}$$ (8B)

When *TPA* or *DTC* is equal to one, the estimated effect on the dependent variable is the multiple of *e* to the corresponding coefficient. For example, in the *Cost* Eq (6B), when *TPA* = 1, farm food safety costs will be $e^{\beta_{2}}$ times what they are when *TPA* = 0.

## Results and discussion

**Fig 1:**
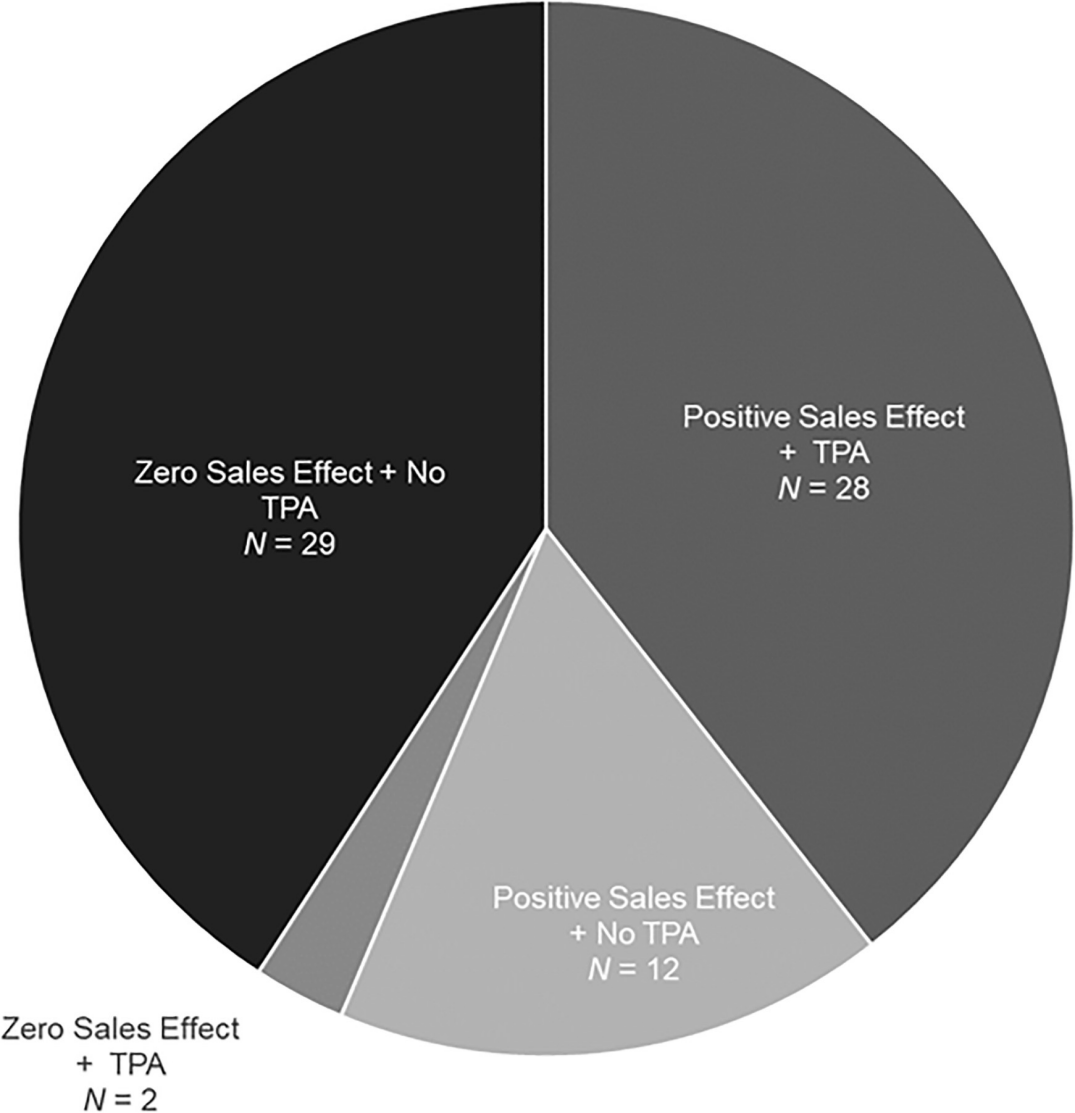
Distribution of farms by food safety improvement sales effect and third party audit occurrence. The number of farms by category include farms that implemented food safety improvements after their GAPS training (n = 71).

Of the 80 farm surveys completed, one farm was excluded in the analysis due to its very small size (0.25 acres) relative to the rest of the sample where the minimum farm size was 2.0 acres. Of the remaining 79 farms, eight of the farms had not implemented any additional food safety practices following the GAPs training and, accordingly, reported zero costs of food safety improvements. Of the remaining 71 farms, only 40 reported positive sales impacts due to their food safety improvements (Fig 1). This may be indicative of new investments based on food safety deficiencies revealed from participating in the GAPs training or in anticipation of future food safety regulatory requirements that have not impacted current sales (e.g., FSMA). Farms are certainly motivated to improve food safety on their own. For example, only 16% of the responding farms completing the GAPs training did so as a buyer requirement. Further, 62% completed a written farm food safety plan after the training, but only 23% did so to meet a buyer requirement. That said, buyer requirements still play an important role. For farms reporting positive sales effects, 70% have had a TPA, compared to 6% for those reporting no sales effects (S2 File).

### Descriptive statistics

The average number of acres in fruits and vegetables was 269, but acreage ranged from 2 to 4,000. Over 50% of the farms had 99 acres or less (Table 1). Total farm acreage was about double that in fruits and vegetables, indicating that multiple farm enterprises were common (e.g., agronomic crops, livestock, agri-tourism, and forestry). Indeed, nearly 30% of the farms had livestock present and 34% were regularly open to the public (e.g., U-Pick operations, Community Supported Agriculture (CSA) farms with work share members, agri-tourism). These characteristics are important as they represent additional food safety risks; i.e., animal manure or run-off into water sources or fields, and risk from additional people present on the farm to serve as vectors for human pathogens.

**Table 1:** Summary statistics of farm respondents.

Variable	Mean	Std Dev	Min	Max
**Continuous variables**				
Number of crops grown	7.30	6.87	1.00	22.00
Farm size, total acres ^a^	524.01	1,177.13	3.80	8,000.00
Farm size, fruit and vegetable acres	269.13	594.84	2.00	4,000.00
**Indicator variables (1 = yes, 0 = no)**				
Farm acres ≤ 15	0.23			
Farm acres 16–99	0.30			
Farm acres 100–499	0.33			
Farm acres ≥ 500	0.14			
Livestock on farm	0.28			
Farm open to public	0.34			
Conducted self audit	0.08			
Had a third party audit (TPA)	0.39			

Mean, standard deviation (Std Dev), minimum (Min) and maximum (Max) are shown for continuous variables, while the proportion of farms in each category are shown for indicator variables.

^a^ One acre is equal to approximately 0.4 hectares (ha).

As expected, smaller-scale farms relied more heavily on DTC markets, such as farm stands, farmers markets, and CSA operations (Table 2). As farm size increased, more reliance on various wholesale buyers were evident, including retail grocery stores, food distributors, and processors. Retailers are particularly prominent in requiring TPAs, in addition to other product requirements for farms in terms of volume, size, and quality.

**Table 2:** Average sales allocation percentages by market channel and farm size.

	Farm size (fruit & vegetable acres) ^a^				
Market channel	All	≤ 15	16–99	100–499	≥ 500
Farm stand/store	18.5	16.8	19.8	22.6	8.7
Farmers market	10.8	18.8	12.6	8.1	0.1
Restaurant, caterer, chef	2.1	1.1	2.9	2.7	0.5
Farm to School program	0.3	0.6	0.6	0.0	0.0
Grocery store	15.8	8.3	11.5	21.5	23.6
Distributor	22.6	5.6	19.0	30.0	40.7
Cooperative	0.8	0.0	2.5	0.0	0.0
Other^b^	29.3	48.9	31.2	15.2	26.4

The sum of sales percentages for each farm size category by market channel equals 100%.

^a^ One acre is equal to approximately 0.4 hectares (ha).

^b^ Common market channels expressed in “Other” included CSA and U-pick operations (smaller farms) and produce auctions and processors (larger farms).

#### Crops produced

**Fig 2:**
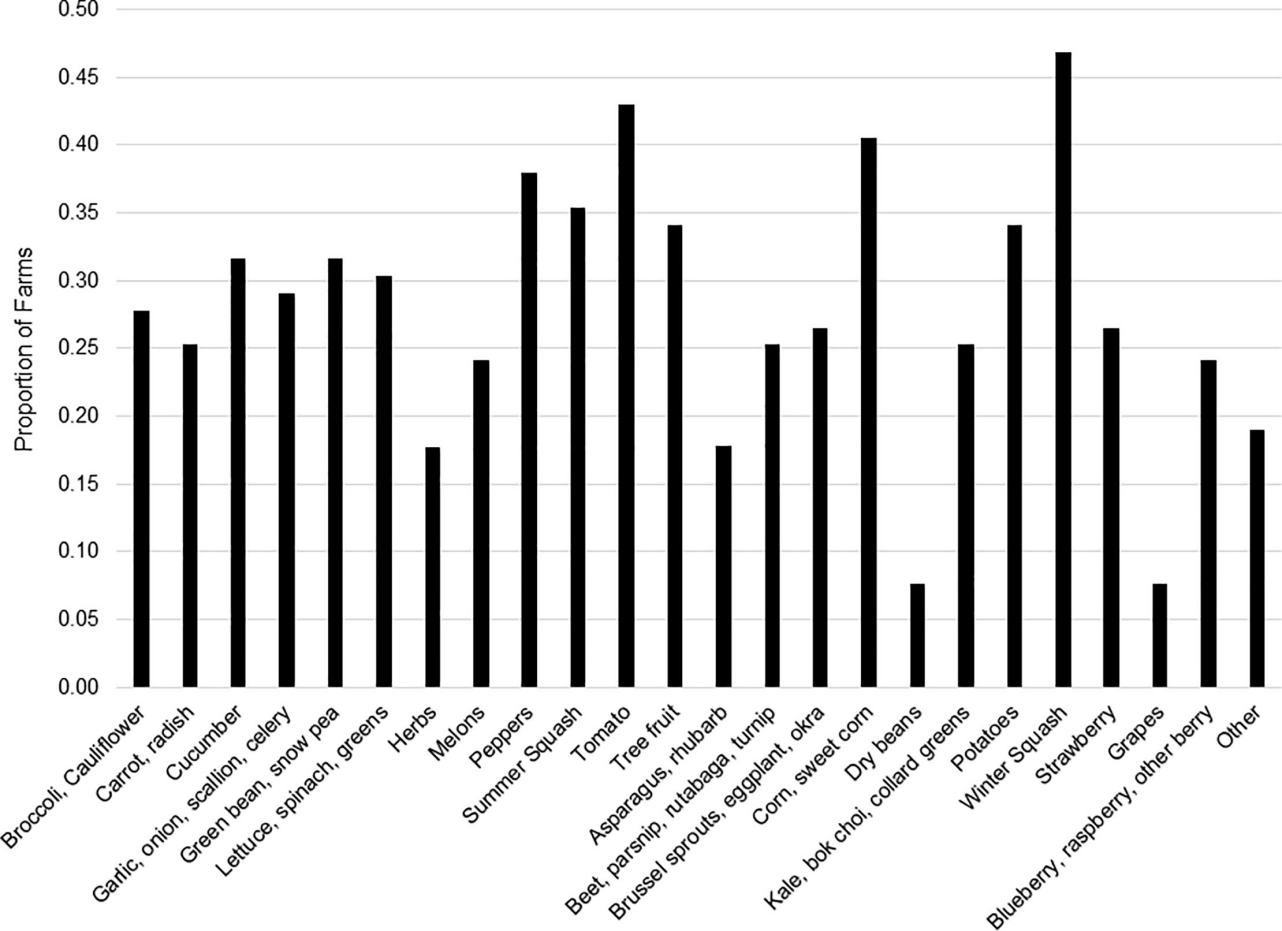
Crops grown by farm respondents, post-GAPs training. Producers selected all categories of crops grown on their farms.

From a list of 24 categories, about seven different crops were grown on the farms, on average, but ranged from only one to as high as 22 (Table 1). Overall, a wide variety of crops were grown and encompassed production through the entire growing season in NYS (Fig 2). In assessing crop planting decisions before and after the GAPs training, there was a very small reduction in the number of crops grown post-GAPs, on average, and statistically indifferent from pre-GAPs levels. Similarly, the average acres in production was statistically unchanged, as were the changes in market channel allocations pre- and post-GAPs.

#### Third party audits (TPAs)

Farms were allowed to select as many types of TPAs that they participated in, with some participating in more than one. Given market channel selection discussed above, it comes as no surprise that larger farms in our sample were more likely to have had TPAs. As shown in Table 3, less than 15% of small farms in our sample (i.e., less than 15 acres in production) had a TPA, while over 60% of the largest farms (i.e., greater than or equal to 500 acres) had. Global GAP TPAs were more common for the larger farm sizes, compared to USDA GAP/GHP TPAs. Several farms also mentioned having commodity-specific or buyer-specific TPAs on their farms.

**Table 3:** Percentages of farms having third party audits, by type and farm size.

	Farm size (fruit & vegetable acres) ^a^				
Type of audit	All	≤ 15	16–99	100–499	≥ 500
USDA GAP/GHP	19.0	11.1	33.3	7.7	27.2
USDA Harmonized GAP	5.1	0.0	8.3	3.8	9.1
Global GAP	13.9	0.0	4.2	26.9	27.2
Commodity specific	19.0	0.0	25.0	19.2	36.4
Buyer specific	1.3	0.0	0.0	3.87	0.0
Other	1.3	5.6	0.0	0.0	0.0
Any	39.2	16.7	50.0	34.6	63.6
None	60.8	83.7	50.0	65.4	36.4

As farms selected multiple audit types depending on their experience, the sum of individual audit percentages for any size category may be larger than the aggregate “Any” category.

^a^ One acre is equal to approximately 0.4 hectares (ha).

#### Reasons for implementing food safety practices

**Fig 3:**
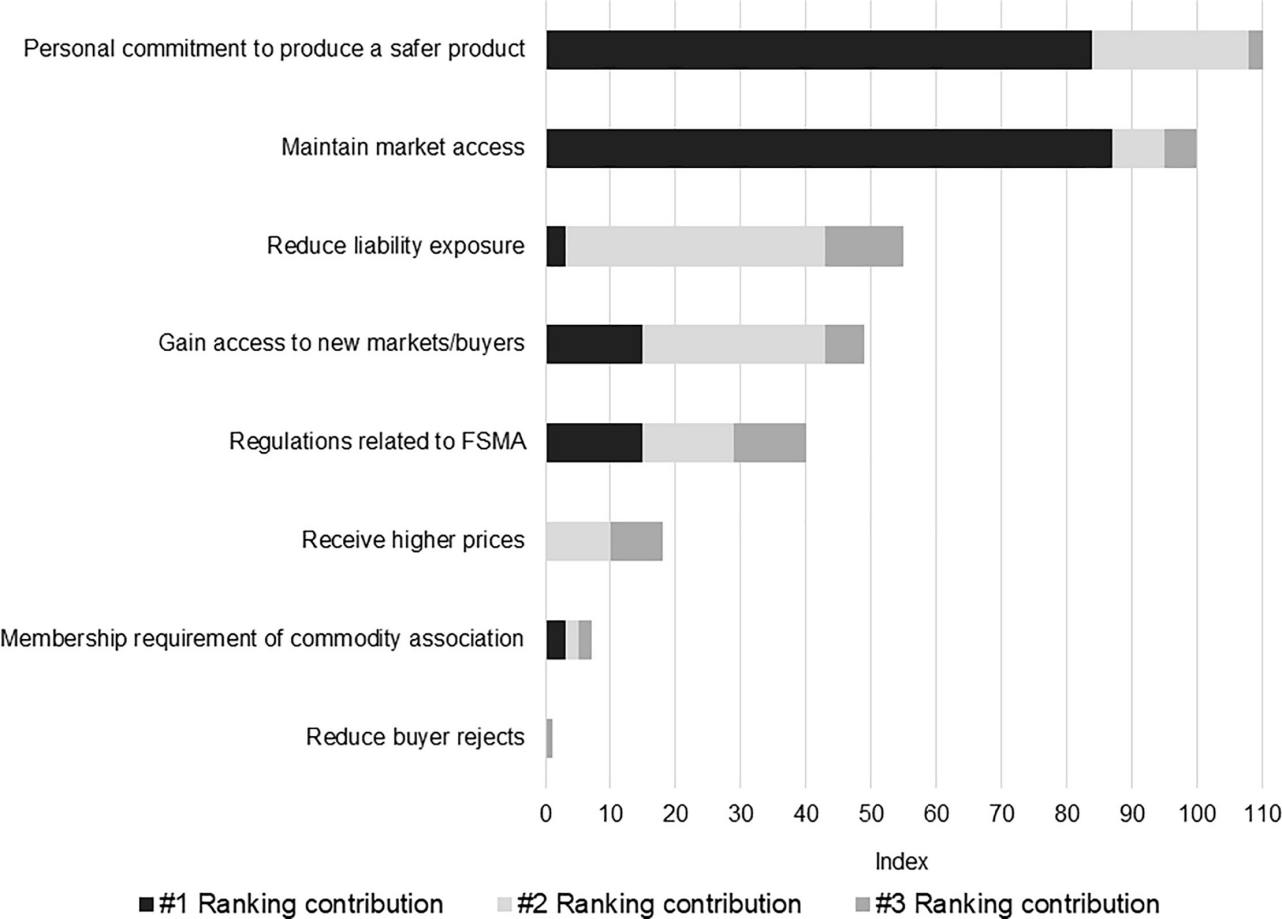
Ranking of reasons for implementing food safety practices. Index scores for each category represents the sum of the number of respondents ranking the category most important times three, the number of respondents ranking the category second most important times two, and the number of respondents ranking the category third most important times one.

Growers ranked the top three reasons why additional food safety practices were implemented on their farms. To assess responses in aggregate, individual responses were scored by their level of rank. Number one ranked items scored 3 points, second ranked items scored 2 points, and third ranked items scored 1 point. Summing points across farms resulted in the overall index scores illustrated in Fig 3.

The highest scoring reason for implementing additional food safety practices was a “Personal commitment to produce a safer product.” This is consistent with actions taken by participants following their training and discussed earlier. Recall, only 16% of respondents completed their GAPs training due to a buyer requirement and, of those that completed a written farm food safety plan, less than 40% did so because of a specific buyer requirement. Put differently, more participants completed written farm food safety plans (62%) than had a TPA (39%) for which it is a prerequisite.

Additional attention to the benefits of food safety investments is reinforced by the farms’ second and fourth ranked responses; i.e., to maintain market access and gain access to new markets and buyers. These, along with reducing farm liability exposure, are ranked higher than motivations driven by new regulatory requirements. Most growers did not have expectations of higher product prices from implementation.

#### Food safety production costs

Costs were broken down into several categories including: (*i*) training costs, (*ii*) implementation costs associated with labor, (*iii*) testing costs, (*iv*) disposable supplies, (*v*) modification costs (e.g., annual capital improvements), and (*vi*) other additional safety costs. Capital investments were converted to an annualized equivalent based on the expected life of the asset and a 5% discount rate. Average food safety costs per acre for implementing GAPs by farm size and by whether the farm had a TPA are shown in Tables 4 and 5, respectively. There is considerable dispersion around the means in both dimensions, due, in part, to differences in initial farm conditions with respect to food safety practices pre-GAPs.

**Table 4:** Average costs and sales per acre from implementation of GAPs food safety practices by farm size.

	Farm Size (fruit & vegetable acres) ^a^				
Category	All	≤ 15 (1)	16–99 (2)	100–499 (3)	≥ 500 (4)
**Costs:**					
Training	17.51	16.68	30.33	10.39	6.08
Implementation Labor	80.44	187.38	91.95	30.48	10.50
Testing	3.31	1.66	7.24	1.60	0.76
Disposable Supplies	35.92	121.52	15.72	15.76	1.73
Modifications	164.44	558.12	70.22	65.19	23.09
Other	10.62	22.78	10.70	7.16	0.82
**Total Costs**	**312.24**	**908.14**	**226.15**	**130.58**	**42.94**
**Significant means-difference tests** ^b^		d2,d3,d4	d1,d4	d1	d1,d2
**Sales Benefits:**					
Maintained	1,260.87	1,260.00	1,491.34	1,223.14	855.60
Expanded	179.67	0.00	374.48	163.48	49.75
**Total Benefits**	**1,440.54**	**1,260.00**	**1,865.82**	**1,386.62**	**905.35**
**Significant means-difference tests** ^b^		None	none	none	none
**Proportion of farms with sales benefits:**					
Maintained sales > 0	0.48	0.13	0.48	0.59	0.73
Expanded sales > 0	0.23	0.00	0.39	0.18	0.27
Either sales > 0	0.56	0.13	0.70	0.59	0.82
**Benefit Cost Ratio**	**4.61**	**1.38**	**8.25**	**10.62**	**21.08**
**Significant means-difference tests** ^b^		d4	none	d4	d1,d3

Averages are computed based on all farms that reported additional food safety investments following the GAPS training, regardless of whether positive sales benefits were reported.

^a^ One acre is equal to approximately 0.4 hectares (ha).

^b^ Means-difference tests across farm sizes were used to compare whether the average totals are statistically different from one another. The null hypothesis is the difference between the two means is zero. d1 indicates that average under consideration is statistically different (at the 95% significance level or less) than the average reported for the first size category. Notations with d2, d3, and d4 are similarly interpreted.

**Table 5:** Average costs and sales per acre from implementation of GAPs food safety practices by third party audit (TPA) occurrence.

Category	All	Without TPA (1)	With TPA (2)
**Costs:**			
Training	17.51	15.11	20.80
Implementation Labor	80.44	93.24	62.95
Testing	3.31	2.55	4.34
Disposable Supplies	35.92	55.41	9.28
Modifications	164.44	232.22	71.79
Other	10.62	2.39	21.87
**Total Costs**	**312.24**	**400.92**	**191.03**
**Significant means-difference tests**^b^		none	none
**Sales Benefits:**			
Maintained	1,260.87	463.15	2,351.08
Expanded	179.67	167.49	196.32
**Total Benefits**	**1,440.54**	**630.64**	**2,547.40**
**Significant means-difference tests**^b^		d2	d1
**Proportion of farms with sales benefits:**			
Maintained sales > 0	0.48	0.20	0.87
Expanded sales > 0	0.23	0.10	0.40
Either sales > 0	0.56	0.29	0.93
**Benefit Cost Ratio**	**4.61**	**1.57**	**13.33**
**Significant means-difference tests**^b^		none	none

Averages are computed based on all farms that reported additional food safety investments following the GAPS training, regardless of whether positive sales benefits were reported.

^a^ One acre is equal to approximately 0.4 hectares (ha).

^b^ Means-difference tests across farm sizes were used to compare whether the average totals are statistically different from one another. The null hypothesis is the difference between the two means is zero. d1 indicates that average with TPA (2) is statistically different (at the 95% significance level or less) than the average reported without TPA (1). If d1 is indicated, by definition, d2 is also indicated.

Food safety improvement costs averaged $312 per acre across the entire sample. While higher than presented in the previous literature [12–16], most previous estimates exclude labor costs and capital investments, which represent nearly 80% of the total costs estimated here. Comparing similar size categories in Sullins and Jablonski [17], our cost estimates are quite similar, providing support to the robustness of our estimates beyond NYS specifically.

Average costs per acre across farm sizes provides preliminary evidence that costs for food safety improvements increase with farm size but less than proportionally. In particular, average costs per acre drop sizably from the smallest ($908) to the largest ($43) farm categories (Table 4). Means-difference tests (that consider the average levels, standard deviations, and sub-sample number of observations) were used to compare whether the computed average total costs per acre are statistically different from one another at the 95% significance level or less. The higher total cost per acre for the smallest farm size category is statistically different than the other three; the difference between the second size category and the largest is also statistically significant. Differences between the second and third categories (*p* value = 0.157) and the third and fourth categories (*p* value = 0.166) were not statistically significant. Notably, the estimated averages by size category ignore any costs differences by TPA experience and marketing channel choices.

Considering differentiation by experience with TPAs, average costs per acre for farms who have had a TPA ($191) are less than half those who have not ($401) (Table 5). However, given the large dispersion across farms around the average estimates, their difference is not statistically significant (*p* value = 0.136). This result is due in large part to the size of farms that have had TPAs (Table 3) and differences in marketing channel choices (Table 2) but without disaggregating these effects.

#### Food safety sales benefits

Average sales impacts from food safety improvements by farm size and by whether the farm had a TPA are also shown in Tables 4 and 5. Fifty-six percent of farms reported either maintained or expanded sales benefits. More farms reported maintained sales benefits (48%) than expanded sales benefits (23%), but both are important. The results vary considerably by farm size, where only 13% of farms in the smallest size category reported benefits compared with 82% in the largest size category (Table 4). As size and TPAs have a strong positive correlation, it is not unexpected that more farms having TPAs reported sales benefits (93%) than those that did not (29%) (Table 5).

Across all farms, food safety improvements resulted in average sales per acre benefits of $1,441, ranging from $905 for the largest farm size category to $1,866 for farms ranging in size from 15 to 99 acres (Table 4). It is not until after 99 acres do average benefits acre reduce with farm size, suggesting that benefits to food safety improvements are relatively higher for smaller-scale farm operations. That said, none of the average benefits are statistically different from one another (the minimum *p* value is between the second and fourth size categories at 0.232). To the degree that the largest farms are also more prevalent in having TPAs, the lower average benefits by farm size are offset by the TPA benefit illustrated in Table 5. Indeed the difference in means for farms that had a TPA with those that did not is highly statistically significant (*p* value = 0.003).

#### Food safety benefit cost ratios

Across all farms, the average BCR was 4.61, implying that the benefits of food safety improvements, on average, were 4.62 times that of their annual cost. Indeed, average BCRs across all farm size categories and TPA status are above one, indicating benefits exceed costs, at least on average.

Average BCRs increase monotonically by farm size category reflecting costs per acre that decline more than their according sales benefits. However, means-difference tests conclude that only the differences between the smallest and largest farm size categories, and between the two largest categories are statistically significant, suggesting substantial dispersion around the mean estimates. For farms with TPAs, lower costs per acre and higher benefits per acre result in a much higher BCR than for those without TPAs (Table 5); however, their difference falls short of significance at the 95% significance level (*p* value = 0.063). Again, the fact that these average estimates do not account for other important farm characteristics clouds the results. For important business and policy implications, isolating the individual impacts by farm size, TPA status, and market channel selection provides a more comprehensive analysis than previously analyzed and to which we turn to next.

### Regression results

It is likely that unobserved factors affect the probability of a grower implementing additional food safety practices after their GAPs training. Recall that eight farm respondents reported no additional food safety improvements following their GAPs training. To account for this, we estimate two-stage Heckman sample selection models for regression Eqs 6A, 7A, and 8A to control for potential section bias [23]. The first-stage selection equation specifies the probability that farm *i* implements additional food safety practices using a probit model and the full sample of farm respondents. In addition to the right-hand-side variables included in Eqs 6A–8A, the selection equation contains three indicator variables likely important to the implementation decision: *Livestock*_*i*_ has a value of one if the farm has livestock and zero if it does not, *Public*_*i*_ has a value of one if the farm is open to the public and zero if it is not, and *SelfAudit*_*i*_ has a value of one if the farm conducted a self audit (no third party) and zero if it did not. These three variables thus serve as instruments to identify selection. An inverse mills ratio (IMR) is computed from the first stage result and is included in the second stage cost, benefit, and BCR models [23].

For ease of exposition to the research objectives, our discussion focuses on the results of the second-stage equations. The probit model results are included in a supplementary information file for the interested reader (S3 File). Notably, the IMR variable in the cost, benefit, and BCR equations (Table 6) is not statistically significant indicating that selection bias is not a concern. Since censoring at zero occurs for the benefit and BCR equations (i.e., some farms report positive costs with zero benefits), these equations were estimated with a tobit model. Since the natural log of zero is undefined, the natural log of benefits for growers reporting zero sales benefits is set to 10^−9^ less than the natural log of the minimum reported positive sales benefit [24; pp. 545–547]. The final results are shown in Table 6. All equations are estimated with maximum likelihood (ML).

**Table 6:** Regression results of cost, sales benefit, and Benefit Cost Ratio (BCR) functions of food safety improvements.

Variable	ln(Cost)		ln(Benefit) ^a^		ln(BCR) ^a^	
Intercept	6.718	***	8.022	***	-0.737	
	(0.811)		(1.140)		(1.612)	
ln(Acres) ^b^	0.411	***	0.450	***	0.302	
	(0.117)		(0.167)		(0.237)	
TPA	0.277		2.170	***	2.672	***
	(0.432)		(0.623)		(0.886)	
DTC	0.042		-1.624	***	-2.051	**
	(0.410)		(0.570)		(0.882)	
IMR ^c^	1.208		0.066		-2.002	
	(1.291)		(1.931)		(2.744)	
Test Ln(Acres) = 1^d^	25.410	***	10.790	***	8.650	***
Log-likelihood	-124.477		-94.170		-107.740	
Pseudo R-squared ^e^	0.210		0.494		0.325	

To account for farms reporting no food safety improvement costs, a Heckman two-stage sample selection model was employed for all equations, where the probability of farms implementing additional food safety practices after the GAPs training is modeled with a probit model. To account for farms reporting positive costs with zero sales benefits, the second-stage benefit and BCR equations account for censoring using a tobit model. All equations are estimated with maximum likelihood (ML). Standard errors in parentheses.

***, **, and * represent statistical significance at the 99%, 95%, and 90% significance levels, respectively.

^a^ Marginal effects are necessary for comparison. Marginal effects for the ln(Benefit) equation for ln(Acres), TPA, and DTC are, respectively, 0.291**, 1.401**, and -1.048**. For the ln(BCR) equation they are 0.194. 1.715**, and -1.316**, respectively.

^b^ One acre is equal to approximately 0.4 hectares (ha).

^c^ IMR = Inverse Mills Ratio, derived from first stage probit model [23]

^d^ Wald statistical tests compare the coefficients on ln(Acres) to one for each equation. In all models, the coefficients are statistically significantly less than one.

^e^ ML models do not produce an R-squared measure. We provide the squared correlation of the actual and predicted dependent variable values as a reasonable proxy.

With respect to the cost equation, the coefficient on ln(Acres) is 0.411, indicating that a 1% increase of acres in production increases food safety costs by 0.411%. The coefficient is statistically different from zero and statistically significantly less than one (as shown in the “Test ln(Acres) = 1” row), indicating that costs vary with farm size but less than proportionally. Our result is consistent with the literature [9, 1–18]. After accounting for farm size effects, the impact on food safety improvement costs by farms primarily marketing through DTC channels and whether the farm has had a 3PA are not statistically significant; i.e., they do not affect costs on their own. This is a reasonable result and consistent with previous work considering marketing channel selection implications for food safety [12, 17].

Given the tobit specification for the ln(Benefit) and ln(BCR) equations, marginal effects, rather than the estimated coefficients, are needed for comparison and interpretation. Marginal effects for the ln(Benefit) equation for ln(Acres), TPA, and DTC are, respectively, 0.291, 1.401, and -1.048, all statistically significant at the 95% significance level. Accordingly, a 1% increase of acres in production increases food safety benefits by 0.291%. As with the cost equation, the coefficient is statistically different from zero and statistically significantly less than one, indicating that the benefits of food safety improvements increase with farm size but, also, less than proportionally. Importantly, this is independent of the influences of TPA participation and primary marketing channel utilization. Indeed, both of these factors are statistically significant. Farms with TPAs have 4.06 (e^1.401^) times the level of food safety benefits as those without, while farms that primarily market through DTC channels have 0.35 (e^-1.048^) times the level of food safety benefits than those that do not.

Since both food safety costs and food safety benefits increased less than proportionally to farm size, understanding the net impact depends on their relative magnitudes. Based on Eq (5) and using the estimated coefficients in Table 6, this would imply 0.450–0.411 = 0.043, a result statistically indifferent from zero (*p* value = 0.207). The estimated coefficient on ln(Acres) in the BCR equation is not statistically different from zero, confirming this result and implying that BCRs for food safety improvements are scale neutral. In other words, the less than proportional increases for food safety improvement costs as farm size grows (an advantage to larger farms) is completely offset with less than proportional increases in food safety improvement benefits (an advantage to smaller farms).

Marginal effects for the ln(BCR) equation for ln(Acres), TPA, and DTC are, respectively, 0.194, 1.715, and -1.316. The marginal effect on ln(Acres) is not statistically significant, while the TPA and DTC coefficients are both statistically significant at the 95% significance level. Given the insignificant results on costs for TPA and DTC and the significant results for these variable on benefits, the relative benefits farms experience with TPAs carries through; i.e., farms with TPAs (independent of farm size) have BCRs 5.56 (e^1.715^) times that of those that do not. Farms that primarily market to DTC channels have BCRs only 0.27 (e^-1.316^) times that of those that do not.

## Conclusions

The multi-day GAPs training program has been an effective way to help growers understand GAPs, how to implement them, develop a written farm food safety plan, and successfully complete a TPA. Growers implement food safety practices on their farm because they are concerned about the safety of the produce they grow and sell. However, beyond a personal commitment to produce safety, farm size, market channel selection, and buyer requirements through TPAs significantly influence the financial impacts of food safety improvements to fruit and vegetable producers.

Our survey of fruit and vegetable farms in NYS suggests that increasing food safety improvements facilitated by GAPs training programs have not significantly impacted the size of farm operations or the types of crops grown. This is consistent with fact that the average estimated financial benefits of food safety investments exceeded the costs associated with their implementation; i.e., BCRs were greater than one. In terms of farm size, we show that both the financial costs and financial benefits of food safety improvements increase with farm size, but at decreasing rates. The net result (our BCR) thereby illustrates that the relatively higher market sales gains by smaller farms in our sample from additional food safety investments offset the relatively higher costs to them of their implementation.

In addition, we clearly demonstrate that benefits of food safety improvements were significantly higher for farms having TPAs and for those that market primarily through non-DTC market channels. Accompanying TPAs are important to the benefits of food safety investments, where farms experiencing them show benefits four times more than those that do not. As food safety investments are not necessarily associated with regulatory nor TPA requirements, we find that farms participating primarily in DTC marketing channels have sales benefits roughly one-third of those focused on non-DTC markets, all else constant. The inherent added value of our approach allows farms to consider the individual financial impacts of food safety investments relative to their farm size, need for TPAs, and market channel selection.

The results presented here should prove welcome by growers to know their food safety efforts are worth the investment and by educators as they encourage participation by all scales of producers in GAPs trainings. This study is also valuable as a benchmark for assessing the implementation of GAPs prior to the FSMA PSR implementation. There is much concern about how the FSMA PSR will impact the economic viability of farms, especially small farms. The results presented in this research indicate that food safety improvements as a result of implementing GAPs may be a signal that overall profitability effects may not differentially impact smaller producers under FSMA PSR to the extent that the costs and types of food safety investments required under FSMA PSR are similar to those defined by our responding farmers. To be sure, the benefits of following through with a TPA after food safety investments are implemented are large, regardless of farm size. Finally, given our relatively small sample of producers in NYS, additional research is needed in assessing food safety costs and benefits across more farms and regions to evaluate the robustness of these results across crops, geographies, and regulatory environments.

## Supporting information

S1 File Grower food safety survey. Grower surveys were administered in person or over the phone, with oral consent provided to the enumerator.
(DOCX)

S2 File Figures for manuscript. An Excel workbook contains the data for the figures used in the manuscript. The figure data can also be recovered from using S1 Code with S1 Data.
(XLSX)

S3 File Probit regression results. To correct for potential selection bias on implementing additional food safety practices following GAPS training, a first-stage probit model was estimated.
(DOCX)

S1 Code SAS code. The analysis uses SAS Version 9.4 to compute descriptive statistics and estimate regression models.
(SAS)

S1 Data Data from grower survey. Data are provided in a SAS data set file.
(SAS7BDAT)

## References

[1] Lynch MF, Tauxe RV, Hedberg CW. The growing burden of foodborne outbreaks due to contaminated fresh produce: risks and opportunities. Epidemiol Infect. 2009;137:307–315. 10.1017/S0950268808001969 19200406

[2] Painter JA, Hoekstra RM, Ayers T, Tauxe RV, Braden CR, Angulo FJ, et al Attribution of foodborne illnesses, hospitalizations, and deaths to food commodities by using outbreak data, United States, 1998–2008. Emerg Infect Dis. 2013;19:407–415. 10.3201/eid1903.111866 23622497 PMC3647642

[3] United States Food and Drug Administration. Federal Register Notice: standards for the growing, harvesting, packing, and holding of produce for human consumption; final rule. 2015. Available from: https://www.gpo.gov/fdsys/pkg/FR-2015-11-27/pdf/2015-28159.pdf.

[4] Beuchat LR. Difficulties in eliminating human pathogenic microorganisms on raw fruits and vegetables. Acta Hortic. 2004;642:151–160. 10.17660/ActaHortic.2004.642.17

[5] Li-Cohen AE, Bruhn CM. Safety of consumer handling of fresh produce from the time of purchase to the plate: A comprehensive consumer survey. J Food Prot. 2002;65(8):1287–1296. 10.4315/0362-028x-65.8.1287 12182482

[6] Ribera LA, Palma MA, Paggi M, Knutson R, Masabni JG, Anciso J. Economic analysis of food safety compliance costs and foodborne illness outbreaks in the United States. Horttechnology. 2012;22:150–156. 10.21273/HORTTECH.22.2.150

[7] Bihn EA, Gravani RB. Role of Good Agricultural Practices in fruit and vegetable safety In: Matthews K.R, editor. Microbiology of fresh produce Washington: ASM Press; 2006; pp. 21–52. 10.1128/9781555817527.ch2

[8] Food and Drug Administration. Guidance for Industry: Guide to minimize microbial food safety hazards for fresh fruits and vegetables. 1998 U.S. Department of Health and Human Services, Washington, DC Available from: https://www.fda.gov/media/117408/download.

[9] Astill G, Minor T, Calvin L, Thornsbury S. Before implementation of the Food Safety Modernization Act’s Produce Rule: A survey of U.S. produce growers. EIB-194. U.S. Department of Agriculture. Economic Research Service, 8 2018 Available from: https://www.ers.usda.gov/webdocs/publications/89721/eib-194.pdf?v=0.

[10] Bovay J, Ferrier P, Zhen C. Estimated costs for fruit and vegetable producers to comply with the Food Safety Modernization Act’s Produce Rule. EIB-195, U.S. Department of Agriculture. Economic Research Service, 8 2018 Available from: https://www.ers.usda.gov/webdocs/publications/89749/eib-195.pdf?v=0.

[11] Astill G, Minor T, Thornsbury S, Calvin L. U.S. produce growers’ decisionmaking under evolving food safety standards. EIB-210, U.S. Department of Agriculture, Economic Research Service 6 2019 Available from: https://www.ers.usda.gov/webdocs/publications/93242/eib-210.pdf?v=5059.4

[12] Hardesty SD, Kusunose Y. Growers’ compliance costs for the Leafy Greens Marketing Agreement and other food safety programs. Research Brief, UC Small Farm Program, University of California-Davis 9 2009 Available from: http://sfp.ucdavis.edu/files/143911.pdf.

[13] Becot FA, Nickerson V, Conner DS, Kolodinsky JM. Costs of food safety certification on fresh produce farms in Vermont. Horttechnology. 2012;22:705–714. 10.21273/HORTTECH.22.5.705

[14] Prenguber B, Gilroy A. A first look at produce safety costs on Oregon’s small and medium fresh fruit and vegetable farms. Oregon Public Health Institute 2013 Available from: https://ophi.org/download/PDF/producesafety_paper_final_OPHI.pdf

[15] Ribera LA, Yamazaki F, Paggi M, Seale JL. The Food Safety Modernization Act and production of specialty crops. Choices. 2016;31(1). Available from: http://www.choicesmagazine.org/choices-magazine/theme-articles/producer-impacts-of-the-food-safety-modernization-act/the-food-safety-modernization-act-and-production-of-specialty-crops.

[16] Lichtenberg E, Page ET. Prevalence and cost of on-farm produce safety measures in the Mid-Atlantic. Food Control. 2016;69:315–323. 10.1016/j.foodcont.2016.04.054

[17] Sullins MJ, Jablonski BBR. What influences produce growers’ on-farm expenditures for food safety? A Colorado investigation of the relationships among farm scale, value of sales, market channel, and expenditure Levels. Western Economics Forum. 2016;15:11–19. 10.22004/ag.econ.253363

[18] Adalja A, Lichtenberg E. Produce growers’ cost of complying with the Food Safety Modernization Act. Food Policy. 2018;74:23–38. 10.1016/j.foodpol.2017.10.005

[19] Bovay J, Sumner DA. Economic effects of the U.S. Food Safety Modernization Act. Appl Econ Perspect Policy. 2018;40:402–420. 10.1093/aepp/ppx039

[20] Tobin D, Thomson J, LaBorde L, Radhakrishna R. Factors affecting growers’ on-farm food safety practices: evaluation findings from Penn State Extension programming. Food Control. 2013;33:73–80. 10.1016/j.foodcont.2013.02.015

[21] Marine SC, Martin DA, Adalja A, Mathew S, Everts KL. Effect of market channel, farm scale, and years in production on mid-Atlantic vegetable producers’ knowledge and implementation of Good Agricultural Practices. Food Control. 2016;59:128–138. 10.1016/j.foodcont.2015.05.024

[22] World Health Organization. Diet, Nutrition and the Prevention of Chronic Diseases. WHO Technical Report Series 916. 2003. Available from: https://www.who.int/dietphysicalactivity/publications/trs916/en/. 12768890

[23] Heckman J.J. Sample Selection Bias as a Specification Error. Econometrica. 1979;47:153–161. 10.2307/1912352

[24] Cameron AC, Trivedi PK. Microeconometrics using Stata. Revised ed College Station: Stata Press; 2010.

