# Supplementary material for: Assessing the costs and returns of on-farm food safety improvements: A survey of Good Agricultural Practices (GAPs) training participants
Source: PLoS One. 2020 Jul 2;15(7):e0235507. doi: 10.1371/journal.pone.0235507 (PMC7332080; doi:10.1371/journal.pone.0235507)
Supplement: S1 File — Grower surveys were administered in person or over the phone, with oral consent provided to the enumerator. (DOCX) [file pone.0235507.s001.docx]

Understanding the Economic Impacts of Implementing Good Agricultural Practices for Produce Safety

Cornell Cooperative Extension would like to help New York produce growers plan for the costs of improving or beginning food safety practices by understanding the cost implications of new produce safety standards and market expectations on the produce sector.

A survey has been developed to assess the costs that fruit and vegetable growers incur when implementing Good Agricultural Practices (GAPs) designed to improve food safety on their farms, during produce processing, and in the markets where they sell their produce. Additionally, this survey will help define the economic impact to fruit and vegetable producers by maintaining existing markets or expanding their business to a new market because of implementing GAPs on their farm.

Answering these questions is voluntary and will take about 20 minutes. Your name and farm information will be kept confidential and will not be connected to your answers in any way since they are not part of our survey and not relevant to the information we are gathering. Any questions that are not relevant to your operation can be skipped.

**To be successful, we need participation from NY fruit and vegetable growers. Thank you** in advance for participating in this survey.

**A. About your farm:** Please answer the following questions about your farm prior to your GAPS training and then changes made to your farm after your GAPS training was completed. If you have not had GAPS training leave the ‘post gaps’ column blank.

**Q1.** Have you completed a GAPs training?

🞏 No 🞏 Yes (if yes, in what year did you complete the training? ____)

🡺 If yes, what type of training?

| Training Type | **Check all that apply** |
| --- | --- |
| Cornell Cooperative Extension 2-day training | 🞏 |
| Wegman’s 1-day training | 🞏 |
| Other | 🞏 |

**Q2.** Acreage planted:

|  | **Pre GAPs** | **Post GAPs** |
| --- | --- | --- |
| Total acres planted in **fruits and vegetables** |  |  |
| Total farm size |  |  |

**Q3.** Crops produced: check all that apply.

|  | **Pre GAPs** | **Post GAPs** |  |  | **Pre GAPs** | **Post GAPs** |
| --- | --- | --- | --- | --- | --- | --- |
| Broccoli, cauliflower |  |  |  | Asparagus, rhubarb |  |  |
| Carrots, radishes |  |  |  | Beets, parsnips, rutabagas, turnips |  |  |
| Cucumber |  |  |  | Brussel sprouts, eggplant, okra |  |  |
| Garlic, onions, scallions, celery |  |  |  | Corn, sweet corn |  |  |
| Green beans, snow peas |  |  |  | Dry beans |  |  |
| Lettuce, spinach, leafy greens |  |  |  | Kale, bok choy, collard greens |  |  |
| Herbs |  |  |  | Potatoes (irish, fingerling, sweet) |  |  |
| Melons (cantaloupe, watermelon, honeydew) |  |  |  | Squash (pumpkin, winter) |  |  |
| Peppers (bell, chile) |  |  |  | Strawberries |  |  |
| Squash (zucchini, summer) |  |  |  | Grapes (table or wine) |  |  |
| Tomatoes |  |  |  | Blueberries, raspberries, other berries |  |  |
| Tree fruit |  |  |  | Other |  |  |

**Q4.** Do you have livestock (ruminants, hogs, poultry) on your farm?

🞏 No 🞏 Yes

**Q5.** Are your farm fields open to the public (U-pick work share or CSA)?

🞏 No 🞏 Yes

**Q6.** Have you ever conducted a self-audit of food safety practices on your farm?

| Pre-GAPs – Yes 🞏 No 🞏 |  |
| --- | --- |
| Post-GAPs – Yes 🞏 No 🞏 |  |

**Q7.** Indicate any third-party food safety audits you have had conducted on your farm:

🞏 USDA GAP/GHP

🞏 USDA Harmonized GAP

🞏 Global GAP

🞏 Commodity-specific __________________

🞏 Buyer-specific ___________________

🞏 Other ________________

🞏 My farm has not had a third-party audit

🡺 If yes, what company/auditing agency conducted the audit? ____________________

**Q8.** Please rank the top three reasons why you implemented food safety practices on your farm:

🞏 Maintain market access (to meet a buyer requirement or preference)

🞏 Regulations related to Food Safety Modernization Act

🞏 Gain access to new markets/buyers

🞏 Meet membership requirement of commodity association

🞏 Personal commitment to produce a safer product

🞏 Reduce liability exposure on the farm

🞏 Reduce chances of buyer rejecting lots/shipments due to spot market tests

🞏 Receive higher product prices

🞏 Other ___________________________________________________

🞏 I have not implemented any food safety practices at this time

**B. Costs of food safety on your farm**

For each question below, as a result of implementing GAPs on your farm please estimate your costs for the growing season. We understand that these may be hard to measure, but provide your best estimate as to hours spent and dollars paid. If some of your costs for food safety are realized by working with a growers’ group, please record your business’s share of those costs.

**Q9. Training Costs:** As a result of implementing GAPs on your farm, estimate the costs of training your workers in basic hygiene practices, monitoring & inspecting crops, handling produce, cleaning tools & equipment, and other tasks related to food safety.

Note that training may include attending a GAPs or other food safety training, organizing and conducting on-farm training for workers and others (e.g. volunteers, part-time labor), paying for someone to translate materials, the time workers spend attending any training.

| Type of staff | **Number of additional hours per season spent on food safety training** | **Number staff trained per season** | **Estimated cost** |
| --- | --- | --- | --- |
| Managers/Owner |  |  |  |
| Field labor |  |  |  |
| Volunteers, interns |  |  |  |
| Other |  |  |  |

**Q10.** **Implementation Costs Associated with Labor:** After implementing GAPs on the farm, estimate how many more hours per week your workers spend on food safety practices such as documenting, monitoring, cleaning and sanitizing equipment, conducting recall exercises, etc., compared to before the implementation of GAPs on your farm.

|  | **Total number of staff with specific food safety duties** | **Number of additional hours per week spent on improved food safety practices** | **Total number of weeks they work during season** | **Workers’ hourly wage rate** |
| --- | --- | --- | --- | --- |
| Managers/Owner |  |  |  |  |
| Paid labor |  |  |  |  |
| Volunteers, interns |  |  |  |  |
| Other |  |  |  |  |

**Q11.** Have you hired any new staff to **develop or** **implement** GAPs on your farm?

🞏 No 🞏 Yes

🡺 If yes, note the number of additional staff hired:

|  | **Number of new staff** |
| --- | --- |
| Managers/owner |  |
| Field labor |  |
| Volunteers, interns |  |
| Other |  |

**Q12. Testing:** As a result of implementing GAPs on the farm, estimate your **additional** costs of testing the safety of your water, soil and soil amendments for the presence of human pathogens or indicator organisms (i.e., quantified generic *E. coli*):

| Input testing | **Number of test locations (each wellhead, spigot, etc. where samples drawn)** | **Number of tests per season** | **Cost per test** | **Related costs (shipping or delivering samples)** |
| --- | --- | --- | --- | --- |
| Irrigation water, what is your water source (well, surface, etc.)________ |  |  |  |  |
| Postharvest water |  |  |  |  |
| Soil |  |  |  |  |
| Soil amendments (composted animal manure, raw manures, agricultural teas, others) |  |  |  |  |
| Other testing ______________________ |  |  |  |  |

Q13. As a result of implementing GAPs on your farm, estimate your additional annual costs for disposable supplies related to food safety on your farm and in your packing and processing areas:

| Items, supplies | Post GAPs |
| --- | --- |
| Hand washing and worker hygiene supplies (soap, paper towels, toilet paper, gloves, first aid supplies) |  |
| Cleaning supplies, detergents, and sanitizers for food contact surfaces (tools, tables, and bins) |  |
| Sanitizers for managing postharvest water quality (chlorine, peroxyacetic acid, peroxide, etc. in a bulk water or single pass situation) |  |
| Monitoring supplies for postharvest sanitizers (ORP meter or pH reader) |  |
| Containers, totes, packaging |  |
| Temperature control supplies (such as thermometers, probes) |  |
| Rodent control supplies (such as traps, bait) |  |
| Office supplies for food safety for traceability program (making posters, labeling tools, software, recordkeeping supplies) |  |
| Recordkeeping supplies (writing instruments, paper, binders, clip boards, white boards, or electronic recordkeeping systems) |  |
| Other |  |

# Q14. Please list additional estimated costs for any modifications you made to improve food safety for production, harvest, processing, or packing as a result of implementing GAPs on the farm:

|  | **Post GAPs** | **Post GAPs** |
| --- | --- | --- |
|  | **One-time investments and improvements** | **Annual estimated maintenance and/or rental** |
| Worker hygiene:  -Toilet facilities (portable toilet rental, construction of new toilet facilities)  -Handwashing station(s)  -Drinking water dispenser(s) |  |  |
| Signage explaining safety policies for workers, volunteers and/or visitors, animal restrictions |  |  |
| Fencing or other deterrents to keep wild or domestic animals out of crop production areas (such as decoys or noise cannons) |  |  |
| Harvesting equipment: (new trailers, sheds) |  |  |
| Packing equipment: (basins, tubs, drying racks, and other structures to improve food safety) |  |  |
| Produce storage & cooling facilities and equipment (walk-ins, room coolers, cooling trailers, hydro cooling, insulation) |  |  |
| Processing/packing costs (cost of services and equipment) |  |  |
| Changes to water source, delivery system or drainage (berm to prevent run-off into source, piping replaced, delivery method – e.g. overhead to drip irrigation) |  |  |
| Changes to raw manure use (composting, building composting facilities) |  |  |
| Other: |  |  |

**Q15.** Estimate any **additional** food safety-related costs on an annual basis:

| Upgrades to website to support food safety education, traceability, signage |  |
| --- | --- |
| New packaging materials and/or labeling (clamshells, bags or other containers, with any food safety messaging) |  |
| Third-party audits |  |
| Insurance (i.e., product liability) |  |
| Other: |  |

Q16. FDA has proposed categorizing all produce farms by their size, based on their total sales of any food for animal and human consumption (this means all fresh produce, processed and packaged foods and animal feed sold on your farm). Please indicate which sales category best fits your farm’s revenues:

| Gross value of sales of food for human and animal consumption from your farm per year | **Pre GAPs** | **Post GAPs** | **2014 season (anticipated upcoming season)** |
| --- | --- | --- | --- |
| Under $25,000 - $100,000 | 🞏 | 🞏 | 🞏 |
| Between $100,001 - $250,000 | 🞏 | 🞏 | 🞏 |
| Between $250,001 - $500,000 | 🞏 | 🞏 | 🞏 |
| Above $500,000 | 🞏 | 🞏 | 🞏 |

**Q17.** Indicate any technical assistance that would help you to better implement food safety on your farm and in your product processing, packing, and marketing (check all that apply).

- Attending GAPs training
- Understanding new produce safety regulations (FSMA)
- Developing a farm food safety plan
- Developing SOPs
- Conducting risk assessments
- Developing/Improving recordkeeping
- Developing/Improving a traceability and recall system
- Testing water for crop production
- Testing postharvest water
- Testing soil or soil amendments
- Managing wildlife with deterrents
- Product storage & handling
- Better communication of food safety to buyers/consumers
- Training resources (managers, labor, volunteers)
- Balancing the management of natural resources (wildlife, water, native plants)
- Other: ______________________________________________

**C. Market impacts of meeting buyer’s food safety requirements**

**Q18.** **Food safety action(s)**: Please indicate which of the following food safe actions you have taken on your farm as a result of implementing GAPs and whether or not they were completed as a requirement by a buyer.

| **Food safety actions** | **Which of the following food safety action(s) have you completed?** | **Which of the following food safety action(s) were completed as a buyer requirement?** |
| --- | --- | --- |
| GAPs Food Safety Training | 🞏 | 🞏 |
| Written Farm Food Safety Plan | 🞏 | 🞏 |
| 3^rd^ Party Audit | 🞏 | 🞏 |

| **Q19.** Indicate the percent of total sales in each market where you sold your products? Fill in all that apply. | | |  |
| --- | --- | --- | --- |
|  | Pre GAPs | Post GAPs | |
| From your farm (farm stand or store) |  |  | |
| Farmers’ market |  |  | |
| Restaurant/caterer/chef |  |  | |
| Farm to school program |  |  | |
| Grocery store |  |  | |
| Distributor |  |  | |
| Co-op |  |  | |
| Other:  _____________________ |  |  | |

**Q20.** As a result of meeting the food safety requirements of your buyer, please indicate what value of markets were maintained or expanded.

|  | **Check all that apply** | **Dollar value of market?** | **Percentage of market?** |
| --- | --- | --- | --- |
| Maintained Sales – (What dollar value and percent of your pre-GAPs sales would have been lost if you did not implement a food safety plan, and thus maintained them as a result of implementing your GAPs food safety plan?) | 🞏 |  |  |
| Expanded Sales – (What dollar value and percent of your post-GAPs sales were the result of increased sales to new or existing markets as a result of implementing your gaps food safety plan?) | 🞏 |  |  |
