# Supplementary material for: Assessing the costs and returns of on-farm food safety improvements: A survey of Good Agricultural Practices (GAPs) training participants
Source: PLoS One. 2020 Jul 2;15(7):e0235507. doi: 10.1371/journal.pone.0235507 (PMC7332080; doi:10.1371/journal.pone.0235507)
Supplement: S3 File — To correct for potential selection bias on implementing additional food safety practices following GAPS training, a first-stage probit model was estimated. (DOCX) [file pone.0235507.s003.docx]

**Supplementary Table 6. Regression Results of First-Stage Probit Model on Probability that Farms Invest in Additional Food Safety Practices following GAPs Training.^a^**

| **Variable** | **TAC_DV** |  |
| --- | --- | --- |
| Intercept | -0.185 |  |
|  | (0.797) |  |
| ln(Acres) | 0.239 |  |
|  | (0.155) |  |
| TPA | 6.342 | *** |
|  | (0.370) |  |
| DTC | -0.075 |  |
|  | (0.695) |  |
| Livestock | 0.922 |  |
|  | (0.706) |  |
| PublicOpen | 0.435 |  |
|  | (0.617) |  |
| SelfAudit | -6.819 | *** |
|  | (0.370) |  |
|  |  |  |
| Log-likelhood | -107.745 |  |
| Likelihood Ratio | 10.200 | *** |
| *N* | 79 |  |
